# Challenges of Preimplantation Genetic Counselling in the Context of Cystic Fibrosis and Other CFTR-Related Disorders: A Monocentric Experience in a Cohort of 92 Couples

**DOI:** 10.3390/genes15070937

**Published:** 2024-07-18

**Authors:** Ugo Sorrentino, Massimo Menegazzo, Ilaria Gabbiato, Davide Calosci, Carlo Federico Zambon, Daniela Zuccarello

**Affiliations:** 1 Clinical Genetics Unit, Department of Women’s and Children’s Health, University of Padova, 35128 Padova, Italy; 2 Kidney and Pancreas Transplant Surgery Unit, Regional Center for Diabetes Therapy, University of Padova, 35128 Padova, Italy; 3 Department of Medicine, University of Padova, 35128 Padova, Italy

**Keywords:** cystic fibrosis, genetic counselling, PGT-M

## Abstract

Cystic fibrosis is a highly prevalent genetic disorder caused by biallelic pathogenic variants in the *CFTR* gene, causing an altered function of the exocrine glands and a subsequent spectrum of hypofunctional and degenerative manifestations. The increasing availability of carrier screening programmes, the enhanced life expectancy of patients due to improved treatment and care strategies and the development of more precise and affordable molecular diagnostic tools have prompted a rise in demand of prenatal diagnosis procedures for at-risk couples, including Preimplantation Genetic Testing (PGT). However, challenges remain: heterogeneity among screening programmes, nuances of variant interpretation and availability of novel treatments demand a considerate and knowledgeable approach to genetic counselling. In this work, we retrospectively evaluated the molecular data of 92 unselected couples who received a diagnosis of CFTR-related status and were referred to the genetics clinic at the University Hospital of Padua for genetic counselling on eligibility for PGT. A total of 50 couples were considered eligible for the procedure based on risk of transmitting biallelic pathogenic variants. We report and discuss our experience with this case series in the context of the Italian medical care system and present an overview of the most relevant issues regarding genetic counselling for PGT in CFTR-related disorders.

## 1. Introduction

Cystic fibrosis (CF; MIM #219700) is the most common life-shortening autosomal recessive disorder in European populations [1], with a carrier frequency as high as 1 in 25 in people of Caucasian origin and an estimated worldwide prevalence of approximately 162,000 diagnosed individuals [2]. The condition is caused by biallelic loss-of-function variants in the *CFTR* gene (Cystic Fibrosis Transmembrane Conductance Regulator; MIM *602421), which encodes a cAMP-dependent chloride channel (CFTR) primarily expressed on the apical membrane of epithelial cells of exocrine ducts, where it regulates water homeostasis and pH concentration [3].

CFTR insufficiency results in obstructed, dilated and ultimately damaged exocrine glands in multiple organs, the most affected being the lungs, pancreas, intrahepatic bile ducts and ejaculatory ducts [4]. Pulmonary involvement manifests early in life with recurrent airway infections causing bronchiectasis, cysts and abscesses with progressive lung tissue fibrosis. Similarly, fibrotic degeneration of pancreatic tissue causes pancreatic insufficiency and subsequent malabsorption of proteins and fats. Pancreatic fibrosis can also affect the endocrine component, resulting in the development of diabetes mellitus. Additionally, patients often show signs of liver damage due to bile duct obstruction, which can progress to biliary cirrhosis. Most affected men are infertile due to unilateral or bilateral vas deferens agenesis, while women may have reduced fertility because of thickened cervical mucus [5,6]. CF can manifest even prenatally as foetal echogenic bowel; in 10% of cases, meconium ileus is the presenting feature at birth. Sweat gland dysfunction has no clinical consequences; however, the “chloride sweat test” is one of the most widely used methods for the diagnosis of CF [7].

CF exhibits marked allelic heterogeneity, as more than 2000 sequence variants have been identified to date [8]. However, not all variants are considered causative of fully expressed CF. Different genotypes can cause variable degrees of CFTR dysfunction, resulting in a diverse range of clinical manifestations. This observation led to the definition of CFTR-related disorders (CFTR-RDs), which comprise all clinical entities associated with CFTR dysfunction that do not fulfil the diagnostic criteria for CF [9]. The most prevalent CFTR-RDs are congenital bilateral absence of the vas deferens (CBAVD), chronic or acute recurrent pancreatitis and disseminated bronchiectasis. In 2022, the European Cystic Fibrosis Society updated the diagnostic criteria for CFTR-RD. In clinical practice, the available data are not always sufficient to ascertain a proper differential diagnosis between a classical form of CF and a CFTR-RD. Such a limit is particularly evident in the setting of prenatal and preconception genetic counselling, where the knowledge of parental variants is the only available information to predict the possible phenotype of the offspring. This is further complicated by the limitations of variant interpretation in the diagnostic setting, especially when whole-gene analysis or carrier screening programmes including variants of controversial classification are involved [10]. Moreover, even when a prediction of classical CF can be made, the progression of pulmonary disease may vary significantly between patients sharing the same variants, as a consequence of modifier genes and environmental factors (infections, diet, treatment administration and smoking) [11,12].

Whenever a couple is identified as at risk of having a child with CF, either prenatal or preimplantation diagnosis can be proposed. PGT-M (Preimplantation Genetic Testing for Monogenic Disorders) enables the identification of genetic alterations in embryos obtained through in vitro fertilization techniques, ensuring that only wildtype- or heterozygous-carrier embryos are transferred to the mother’s uterus. According to the most recent data from the European Society of Human Reproduction and Embryology (ESHRE), preimplantation analysis for CF was the fifth most frequent indication for PGT-M and the most common among recessive disorders [13]. However, it is noteworthy that the prevalent approach in scientific literature and clinical practice suggests refraining from prenatal and preimplantation diagnosis when the clinical significance of parental *CFTR* variants is unknown or when these variants are known to be not causative of the classic form of CF [14].

In this study, we retrospectively evaluated the clinical and molecular data of a cohort of 92 at-risk couples who were referred for genetic counselling with questions regarding whether to undergo PGT procedure, assessing their eligibility based on the characteristics of their *CFTR* genotype according to updated guidelines and variant classification resources. We then report our experience in the context of the Italian medical care system and discuss the most relevant issues regarding genetic counselling for Preimplantation Genetic Testing in CFTR-RDs.

## 2. Materials and Methods

### 2.1. Patients

This study retrospectively evaluated a case series of couples referred to the Clinical Genetics Unit of the University Hospital of Padova for genetic counselling for having a presumably higher risk of transmitting CF or a CFTR-RD, with a specific focus on their eligibility for PGT-M procedures. The case series covers the period from January 2016 to December 2022 and includes 92 couples with a total of 184 subjects, 92 males and 92 females. The mean male age was 37 and the mean female age was 34. Relevant written informed consent was obtained from participants or their legal representatives. This study adheres to the principles expressed in the Declaration of Helsinki.

### 2.2. Molecular Analyses and Variant Interpretation

Couples in this study were referred to our clinic after receiving molecular diagnosis of their CFTR status from various external or affiliated laboratories, each using their own variant panels and molecular techniques. All *CFTR* variants (Reference Sequence: NM_000492.4) reported in the patients were reassessed based on ACMG/AMP criteria [15] and by cross-referencing with the CFTR2 (https://cftr2.org/, last accessed on 13 December 2023), CFTR-France (https://cftr.iurc.montp.inserm.fr/cftr, last accessed on 13 December 2023) and ClinVar variation databases (last accessed on 13 December 2023), as well as with data from the existing literature (pertinent English language articles were searched in PubMed, https://pubmed.ncbi.nlm.nih.gov/, last accessed on 13 December 2023). In the article text, all variants were reported according to HGVS nomenclature [16]; when available, the traditional (‘legacy’) names of the variants have also been listed in the tables. The prediction of phenotypes of the possible offspring in cases where they received variant alleles from both parents were estimated using the CFTR2 database and evidence from the literature.

Partners of CF-causing variant carriers in whom second-carrier status could not be diagnosed—or in whom it was not confirmed after the reassessment—were offered second- and third-level CFTR investigations by means of Next-Generation Sequencing (NGS) exome sequencing and Multiplex Ligation-dependent Probe Amplification (MLPA), if not already performed and if eligible according to national guidelines [17]. NGS analyses were performed on genomic DNA extracted from peripheral leukocytes. The coding regions of the selected gene were isolated and captured using the SureSelect Target Enrichment systems (Agilent Technologies, Santa Clara, CA, USA); indexed DNA fragments libraries were generated according to the manufacturer’s protocol and sequenced on a NextSeq 550 instrument (Illumina, San Diego, CA, USA). Variant calling and annotation, as well as a subsequent bioinformatic analysis to detect copy number variations, were performed using the SureCall software v.4.2.2.3 (Agilent Technologies). A panel of known pathogenic deep intronic variants was also detected.

MLPA analyses were performed using SALSA MLPA kit P091 (MRC Holland, Amsterdam, The Netherlands) following the manufacturer’s protocol. Variant frequency in the general population was extracted from the Genome Aggregation Database (gnomAD v.3.1.2, last accessed on 13 December 2023).

## 3. Results

Analyses performed in the 184 subjects of the study cohort revealed 51 different sequence variants in the *CFTR* gene, of which 37 were exonic and 14 intronic (including poly-T tract variants). Out of 51 variants, 34 (66.7%) were classified as CF-causing or associated with variable clinical consequences (VCC) or CFTR-RD (Table 1). The most prevalent were c.1521_1523delCTT p.(Phe508del) (31.25% of variant alleles), c.3484C>T p.(Arg1162*) (5%), c.1624G>T p.(Gly542*) (5%), c.2051_2052delAAinsG p.(Lys684Thrfs*4) (5%), c.3909C>G p.(Asn1303Lys) (4.38%), c.3454G>C p.(Asp1152His) (3.13%) and c.489+3A>G p.(?) (3.13%). As expected, the variants were comparably distributed in male and female subjects, with the exception of poly-T tract variants that were more prevalent in male subjects due to selection bias for infertility screening. The frequencies of the individual variants were comparable to those described in other works in the literature [18,19].

**Figure 1:**
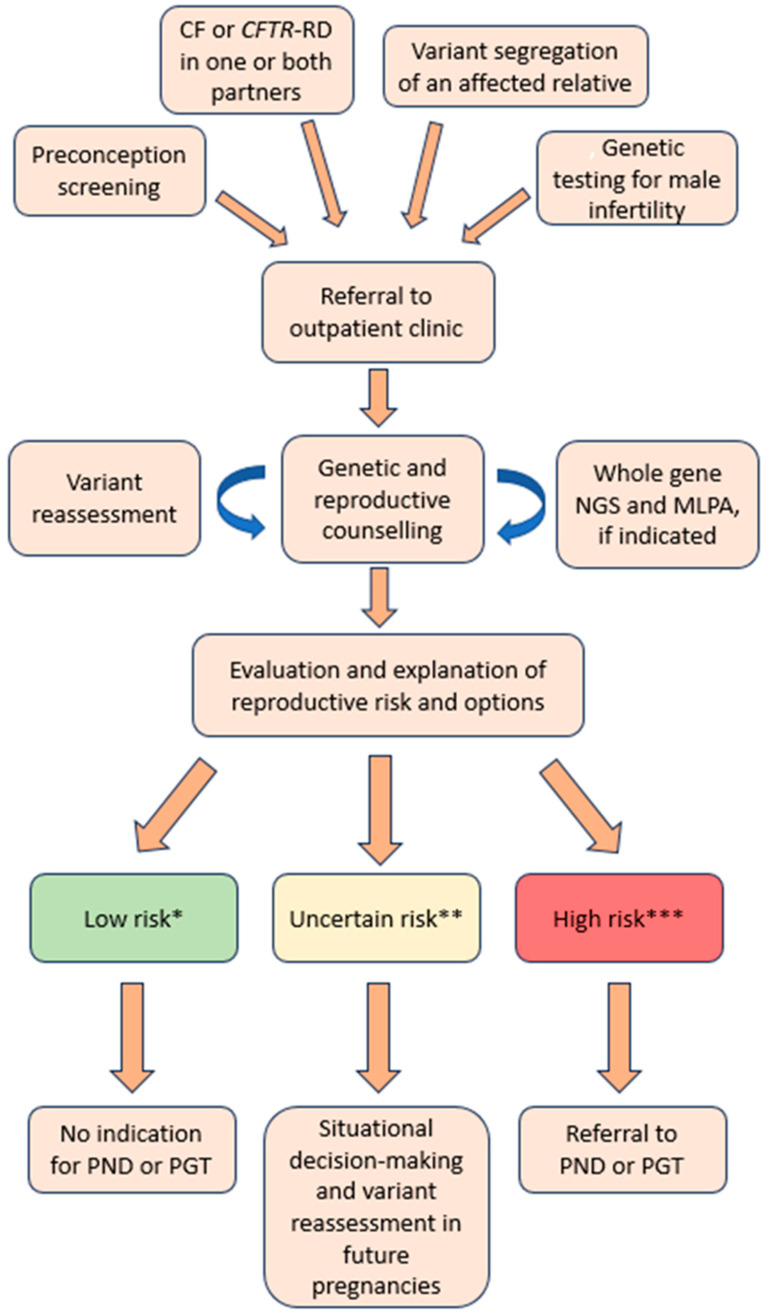
Genetic counselling flow diagram for couples referred on suspicion of being carriers of *CFTR* pathogenic variants. *: couples carrying only one pathogenic variant allele or none. **: couples carrying one pathogenic variant allele and one VUS allele or carrying two VUS alleles. ***: couples at risk of transmitting two pathogenic variant alleles with a predicted phenotype of cystic fibrosis or CFTR-RD not limited to CBAVD. CF: cystic fibrosis. PND: prenatal diagnosis. PGT: prenatal genetic testing.

As a general rule for clinicians at our centre, couples were considered eligible for PGT-M when both partners carried one or more variants whose combination was predicted to be associated with CF or CFTR-RD not limited to CBAVD, as prescribed by national guidelines [17]. A flow diagram of the counselling process is represented in Figure 1. Of the 92 couples who received genetic counselling in the observed time frame, 50 met the criteria to be proposed for PGT-M (Table 2). In 11 couples, the available evidence was insufficient to predict a phenotype clearly associated with the disease and the eligibility for the procedure was discussed on an individual basis taking into account the patients’ risk perception and general reproductive history. For the remaining 31 couples, the predicted combination of variants, or lack thereof in the couples with just a single identified variant allele, did not meet the criteria for sensible use of the procedure. In 23 (46%) of the 50 couples who were considered eligible for PGT-M, at least one partner carried the c.1521_1523delCTT p.(Phe508del) variant and both partners in eight cases (16%). In addition to PGT-M, six couples were referred to PGT for aneuploidy (PGT-A) as well and two more to PGT for structural rearrangements (PGT-SR), due to the presence of a balanced chromosomal abnormality in one partner or advanced maternal age.

To complement the information given to couples during genetic counselling, we investigated whether any combination of carried variants could benefit, in a hypothetical affected offspring, from pharmacological therapy with CFTR modulators (elexacaftor, tezacaftor, ivacaftor and lumacaftor) [20]. Consultation of CFTR2 and CFTR-France variant databases revealed that any affected offspring of 39 (64%) out of 61 couples with high or undetermined risk of disease transmission from our series would be able to benefit from the main drugs currently available. The results are reported in Table 3.

In the 42 couples who did not receive a clear indication to preimplantation diagnosis, two main categories could be identified: those in which at the end of the diagnostic process only one individual (or neither) had been confirmed as a carrier, thus resulting in a low risk of recurrence (25 out of 42) or those in which variants of uncertain significance (VUS), benign variants, or benign of poly-T, TG repeats were identified in either or both partners, entailing either insufficient reliability in predicting the phenotype of a possible compound heterozygous progeny or low risk of transmission of the disease (Table 2). The majority of these patients had been referred for genetic counselling after having directly undergone the second-level genetic examination, which resulted in the report of a VUS, or after having been screened for couple infertility, which, in contrast to the current national guidelines for preconception screening [17], does include the report of poly-T, TG repeats. It should be noted that, despite being informed that their risk was undetermined and therefore PGT-M could not be advised in their cases, most couples carrying a pathogenic variant and a VUS, or even two VUS, expressed a firm intention of proceeding with PGT-M anyway, often supported by the idea that they already embarked on an ART programme and could therefore add the analysis to the procedure without excessive additional burden. A remarkable example of this was couple 61, who was at risk of having an offspring with compound heterozygosity for a pathogenic variant and the [1210-34TG[14];1210-12T[5]] repeat, resulting in a phenotype of uncertain prediction. Because they were already on an ART programme for idiopathic infertility including a PGT-A for maternal age, after thorough discussion, an option was given to add the PGT-M for CFTR, postponing the decision regarding embryo selection to a time when viable embryos could be estimated. Another rather specific circumstance, although concerningly common compared to the total size of our cohort, was raised by couples who had previously experienced voluntary termination of pregnancy in other clinics, due to the prenatal diagnosis of a foetus carrying variant combinations of uncertain significance, as for couple 52, or combinations associated with low risk due to the presence of a putatively benign variant, as for couples 62, 63, 64. Despite receiving a revised and updated genetic assessment, these couples also expressed a strong determination to turn to external PGT centres to perform the procedure rather than finding themselves in a situation similar to what they experienced the past.

Regarding post-consultation follow-up, sixteen couples have so far performed at least one cycle of ART with PGT-M. Of these, eight have been diagnosed with wildtype- or healthy-carrier embryos eligible for transfer, resulting in the birth of unaffected children. In the eight couples that did not experience reproductive success, there were two cases of biochemical pregnancy, three cases of no blastocysts suitable for transfer and three cases of failed embryo implantation post-transfer.

## 4. Discussion

Preimplantation diagnosis, as opposed to prenatal diagnosis, allows couples at risk of transmitting a genetic disease to know the genetic status of their offspring before they become pregnant, thus averting the need to postpone their reproductive choices to a later gestational age. CF is among the most prevalent inheritable genetic disorders and was also the first monogenic condition diagnosed by PGT in 1992 [21]. Since then, several experiences have been reported, contributing to defining, and redefining over time, the most relevant issues and strategies of this complex procedure [14,18,19,22]. To date, several techniques are available for performing PGT-M, including the use of NGS technologies [23,24], making the range of available services increasingly broader and more accessible for couples carrying a monogenic disease.

Our study reports a cohort of 92 couples consisting of healthy CFTR carriers or CF patients, who were referred to genetic counselling to assess their reproductive risk in the perspective of a PGT-M procedure. Of these couples, 50 were considered eligible, as the combination of variants identified in the two partners was predicted to cause a CF or CFTR-RD phenotype with significant impact on pancreatic function or respiratory capacity. However, it must be emphasised that defining the reproductive risk of carriers, regardless of the availability of national and international guidelines and increasingly comprehensive variant databases, is not always an automatic process and requires great care in considering all relevant molecular and clinical aspects of the couple.

Among the most relevant information that a clinical geneticist must consider when counselling CFTR at-risk couples is the clinical context in which their risk has been identified or suspected. Overall, CFTR screening and diagnostic strategies have led to the identification of a large pool of carriers, requiring genetic counselling to evaluate their effective reproductive risk and available choices. The main situations that can lead to identification of carrier status can be through i. preconception screening; ii. one or both partners having been diagnosed with CF or CFTR-RD or having a previous affected pregnancy; iii. variant segregation of an affected relative; and iv. genetic testing for male infertility [17]. While confirmed diagnoses in a patient or affected relative allow, most of the times, for a straightforward interpretation of the causative genetic variants and their associated risk, counselling can be considerably more complex whenever presumed carriers with no previous family history of disease are involved. In the setting of our clinic, the referral of most at-risk couples can be traced back to preconception carrier screening. Specifically, due to the high frequency of mutated alleles in its general population, legislators from the Italian region where our outpatient clinic operates have implemented CFTR screening programmes as part of the granted preconception medical care for couples. Over the years and in different laboratories, many customized, commercially available variant panels have been used, with the selection of investigated variants often left at the discretion of the providers. This issue was addressed in 2019 by the Italian Genetics Society (SIGU), which provided stricter guidelines for CFTR screening programmes and diagnostic investigations [17]. In particular, it was established that first-level screening for carriers should include all pathogenic variants with a relative frequency of 1% or higher and amounting altogether to at least 85% of known causative variants of the geographical area and ethnicity of the individual under consideration and that, in general, a residual risk for the couple equal or lower to 1/777 (0.13%) should be considered sufficient for not proceeding with further investigations. Indeed, second (whole-gene NGS)- and third (MLPA)-level analyses should be carried out only in specific circumstances, such as uncommon ethnicity for that area or in the partner of an affected individual [17]. However, many challenges remain, as prescriptive uniformity has not yet been achieved and several first-level screening panels still contain variants of dubious pathogenicity or variable clinical consequences, making it mandatory for the genetic consultant to always reassess the pathogenicity criteria of the identified variants.

Similar issues can be experienced when assisting a different pool of consultants, who are those referred in the context of infertility diagnostic investigations which often include CFTR analyses, especially considering that CFTR heterozygous carriers have a slightly higher probability of being infertile themselves [25] and even more so if a clinical diagnosis of CBAVD has already been made. Predictably, in this subset of patients, the identification of *CFTR* variants associated with CFTR-RD limited to male infertility is considerably more frequent than in the general population. This can raise issues of appropriateness of PGT-M due to the relatively mild and late onset of the predicted disorder. Particularly relevant to this with regards to male infertility are the c.[1210-34TG[m];1210-12T[n]] polymorphisms of the poly-thymine (poly-T) tract and its modulating TG repeats in intron 8 of *CFTR*, which are a mandatory search in CBAVD cases [26]. The TG12T5 and TG13T5 pathogenic haplotype variants are associated mostly with single-organ involvement, milder symptoms, variable expressivity, and reduced penetrance [27]. For such reason, PGT-M for this category of variants is discouraged by Italian national guidelines [17]. However, the complex integration of counselling by clinicians and decision-making by patients cannot always be as straightforward as recommended by the general rule, especially if more uncommon situations arise. A fitting example of this is couple 61 in our cohort, who had a 25% recurrence risk of compound heterozygosity for a pathogenic variant and the uncommonly reported TG14T5 allele, which is associated with a less predictable risk of disease. With regards to decision-making and counselling in such borderline situations, it is important to remember that these infertile couples are often already in the process of undergoing an ART procedure, posing them with a double-edged dilemma: on one side, it may be seen as an opportunity for performing additional tests and selecting the best possible embryos in the context of an already-ongoing implantation treatment; on the other, this reduces the number of embryos available for transfer, potentially proving detrimental to the procedure’s chances of success. Thorough evaluation of the ovarian reserve and prioritization of PGT-A should be considered to support the best possible choice.

Similarly to hypomorphic variants and variants associated with variable consequences, the identification of a VUS during carrier screening or in-depth NGS-based diagnostics represents one of the most critical occurrences in risk evaluation of inheritable diseases, both from the patient’s and the physician’s perspective [28]. The main problem with VUS is that their identification does not allow precise prediction of pathogenicity; this is even more critical in the preconception and prenatal settings, where it results in the inability to provide couples with reliable estimates of the unborn child’s risk of disease. For this reason, excessively enriched screening panels including recurrent VUS or prescription of whole-gene NGS analyses without precise indication should be considered inappropriate and avoided, as the results may leave patients and clinicians into uncertainty leading to potential errors of risk assessment and perception. Paradoxically, the fact that the *CFTR* gene and its variants are among the most-studied and most-reported genetic diseases, with large dedicated national and international databases, can sometimes be a disadvantage rather than an advantage in terms of variant interpretation. Indeed, the available online databases often contain incomplete information, allele frequencies that are outdated or influenced by selection bias, or even discordant pathogenicity classifications, giving rise to misleading or conflicting interpretations of possible clinical consequences. In our cohort, in situations where the uncertainty had not been resolved, in accordance with national guidelines and after careful counselling, couples were advised that PGT-M could not be granted by the Italian Healthcare System because the main reason for negative embryo selection for this variant combination was missing. However, most of these patients’ perceptions of risk had already been conditioned by the time the variant was reported or by the outcome of previous pregnancies; thus, they felt it was more acceptable to undergo a PGT programme rather than remain in uncertainty. Regardless of the final decision about the procedure, couples who had an uncertain risk profile at the time of consultation were always invited to return to counselling in the event of planning a new pregnancy, in order to assess whether their risk had changed in the meantime due to the emergence of new scientific evidence.

A further element of increasing relevance in genetic counselling for CF and CFTR-RD reproductive risk, and which may influence the couple’s choice on whether to embark on a PGT programme, is the increasing availability of therapeutic options, particularly pharmacological ones [20]. These aspects must be clearly explained in counselling to provide couples with as complete a perspective as possible of the options available to them and their unborn child. In our case series, more than half the couples had a combination of variants such that some degree of efficacy of drugs belonging to the CFTR modulators category could be expected. This information can have major consequences for the reproductive choices of future parents and should therefore be communicated and discussed.

In conclusion, navigating the pitfalls and specificities of genetic counselling of at-risk CFTR couples remains a complex effort, even after decades of international PGT-M experience for CF and CFTR-RD. The implementation of national and international guidelines, the correct calibration of population-based carrier screening panels, careful prescription of NGS-based analyses and the harmonization of online variant databases are all steps in the right direction in allowing the clinical consultant to provide the best possible service. However, the complexity and sensitivity of the information and decisions at stake, including the ethical and personal expectations of the couple, always maintain the need to keep counselling not only as a place for the automatic application of criteria and guidelines, but also for open discussion with the consultees. To better help couples with their decision, proper information about available postnatal treatments for the disease should also be discussed.

## Figures and Tables

**Table 1:** List of identified variants and their functional classifications according to CFTR-France and CFTR2.

Coding Sequence Variant	Protein Sequence Variant	Traditional (“Legacy”) Name	Number of Variant Alleles	Sex	Percentage of Variant Alleles	Classification (Cftr-France)	Classification (Cftr2)	Final Comment at Counselling	Gnomad 3.1.2 Europeans	Gnomad 3.1.2 Global
c.1521_1523delCTT	p.(Phe508del)	F508del	50	23 M; 27 F	31.25%	DC-CF causing	CF causing	CF causing	0.01494	0.01193
c.2051_2052delAAinsG	p.(Lys684Thrfs*4)	K684SfsTer38	8	5 M; 3 F	5.00%	DC-CF causing	CF causing	CF causing	NF	NF
c.3484C>T	p.(Arg1162*)	R1162X	8	4 M; 4 F	5.00%	DC-CF causing	CF causing	CF causing	0.00003307	0.00004588
c.1624G>T	p.(Gly542*)	G542X	8	4 M; 4 F	5.00%	DC-CF causing	CF causing	CF causing	0.0004039	0.0003629
c.3909C>G	p.(Asn1303Lys)	N1303K	7	4 M; 3 F	4.38%	DC-CF causing	CF causing	CF causing	0.0001756	0.0001577
c.3454G>C	p.(Asp1152His)	D1152H	5	5 F	3.13%	DC-VCC	VCC	VCC	0.0004172	0.0003981
c.489+3A>G	p.(?)		5	3 M; 2 F	3.13%	DC-CFTR-RD causing	VCC	VCC	0.0002080	0.0001776
c.2657+5G>A	p.(?)	2789+5G>A	4	2 M, 2 F	2.50%	DC-VCC	CF causing	CF causing	0.00009604	0.00007825
c.1040G>C	p.(Arg347Pro)	R347P	3	2 M; 1 F	1.88%	DC-CF causing	CF causing	CF causing	0.00005255	0.00003966
c.1585-1G>A	p.(?)		3	2 M; 1 F	1.88%	DC-CF causing	CF causing	CF causing	0.0001853	0.0001412
c.3718-2477C>T	p.(?)		3	3 M	1.88%	DC-VCC	CF causing	VCC	0.00008827	0.0001184
c.1584G>A	p.(=)	E528=	3	1 M; 2 F	1.88%	Non DC	Non CF causing	Non DC	0.02363	0.02008
c.579+3A>G	p.(?)		2	2 M	1.25%	DC-VCC	CF causing	VCC	0.00007214	0.00005358
c.3485G>T	p.(Arg1162Leu)	R1162L	2	1 M; 1 F	1.25%	DC-CFTR-RD causing	Non CF-causing	CFTR-RD	0.0009971	0.0007885
c.3230T>C	p.(Leu1077Pro)	L1077P	2	2 F	1.25%	DC-CF causing	CF-causing	CF causing	0.000003391	0.000003099
c.350G>A	p.(Arg117His)	R117H	2	1 M; 1 F	1.25%	DC-CFTR-RD causing	VCC	VCC	0.002710	0.002135
c.1585-9412A>G	p.(?)		2	1 M; 1 F	1.25%	DC-CF causing	NF	LP - CF causing	NF	NF
c.224G>A	p.(Arg75Gln)	R75Q	2	1 M; 1 F	1.25%	Non DC	Non CF causing	Non DC	0.03832	0.03008
c.274-1G>C	p.(?)		2	1 M; 1 F	1.25%	DC-CF causing	CF causing	CF causing	NF	NF
c.3846G>A	p.(Trp1282*)	W1282X	2	2 F	1.25%	DC-CF causing	CF causing	CF causing	0.00007629	0.0002609
c.328G>A	p.(Asp110Asn)	D110N	1	1 M	0.63%	NF	NF	VUS	NF	NF
c.601G>A	p.(Val201Met)	V201M	1	1 F	0.63%	DC-CFTR-RD causing	US	CFTR-RD	0.0001068	0.0001685
c.[601G>A;1210-12T[5]]	p.(Val201Met)	V201M	1	1 F	0.63%	DC-CFTR-RD causing	US	CFTR-RD	0.0001068	0.0001685
c.2787C>T	p.(=)	T990=	1	1 F	0.63%	NF	NF	VUS	NF	NF
c.961T>C	p.(Ser321Pro)	S321P	1	1 F	0.63%	NF	NF	VUS	0.000002856	0.000001591
c.2249C>T	p.(Pro750Leu)	P750L	1	1 M	0.63%	DC-CFTR-RD causing	VCC	VCC	0.0007809	0.0006038
c.1046C>T	p.(Ala349Val)	A349V	1	1 F	0.63%	Unclassified-VUS 4	US	VUS	0.0003687	0.0002919
c.317T>C	p.(Ile106Thr)	I106T	1	1 F	0.63%	NF	NF	VUS	0.000005397	0.000004105
c.3302T>A	p.(Met1101Lys)	M1101K	1	1 F	0.63%	DC-CF causing	CF-causing	CF causing	0.00001436	0.000007689
c.1766+1G>A	p.(?)		1	1 M	0.63%	DC-CF causing	CF-causing	CF causing	0.0001393	0.0001037
c.1392+3A>G	p.(?)		1	1 F	0.63%	NF	NF	VUS	0.000002886	0.000003212
c.2900T>C	p.(Leu967Ser)	L967S	1	1 M	0.63%	Unclassified-VUS 2	VCC	VUS	0.001661	0.001351
c.2052dup	p.(Gln685Thrfs*4)	2184insA	1	1 F	0.63%	DC-CF causing	CF causing	CF causing	0.00003899	0.00003409
c.2463_2464delTG	p.(Ser821Argfs*4)	2594delGT	1	1 F	0.63%	NF	CF causing	CF causing	NF	NF
c.3889dup	p.(Ser1297Phefs*5)	4016insT	1	1 F	0.63%	DC-CF causing	CF causing	CF causing	0.00002469	0.00002045
c.2856G>A	p.(Met952Ile)	M952I	1	1 M	0.63%	DC-CFTR-RD causing	NF	CFTR-RD	0	0.00001710
c.254G>A	p.(Gly85Glu)	G85E	1	1 F	0.63%	DC-CF causing	CF causing	CF causing	0.00008435	0.00006338
c.1163C>T	p.(Thr388Met)	T388M	1	1 M	0.63%	Unclassified-Undefined	NF	VUS	0.00008463	0.0001154
c.3908delA	p.(Asn1303Thrfs*25)	4040delA	1	1 M	0.63%	NF	CF causing	CF causing	NF	NF
c.3854C>A	p.(Ala1285Asp)	A1285D	1	1 M	0.63%	NF	NF	VUS	NF	NF
c.125C>T	p.(Ser42Phe)	S42F	1	1 M	0.63%	Unclassified-VUS 2	NF	VUS	0.0001701	0.0001287
c.316A>T	p.(Ile106Leu)	I106L	1	1 F	0.63%	NF	NF	VUS	NF	NF
c.2654G>C	p.(Gly885Ala)	G885A	1	1 M	0.63%	NF	NF	VUS	NF	NF
c.4426C>T	p.(Gln1476*)	Q1476X	1	1 F	0.63%	DC-CFTR-RD causing	VCC	VCC	0.00002712	0.00002045
c.2991G>C	p.(Leu997Phe)	L997F	1	1 M	0.63%	DC-CFTR-RD causing	Non CF causing	CFTR-RD	0.001733	0.001822
c.3154T>G	p.(Phe1052Val)	F1052V	1	1 M	0.63%	DC-CFTR-RD causing	VCC	VCC	0.0004704	0.0004637
c.1209+28C>T	p.(?)	1341+28C>T	1	1 M	0.63%	Unclassified	NF	VUS	0.0002848	0.0002490
c.3194T>C	p.(Leu1065Pro)	L1065P	1	1 M	0.63%	DC-CF causing	CF causing	CF causing	0.000004497	0.000003421
c.1210-34TG[10];1210-12T[5]	p.(=)	TG10T5	5	5 M	3.13%	NF	NF	Non DC	Not relevant	Not relevant
c.1210-34TG[12];1210-12T[5]	p.(=)	TG12T5	1	1 F	0.63%	DC-CFTR-RD causing	VCC	CFTR-RD	Not relevant	Not relevant
c.1210-34TG[13];1210-12T[5]	p.(=)	TG13T5	1	1 F	0.63%	DC-VCC	VCC	VCC	Not relevant	Not relevant
c.1210-34TG[14];1210-12T[5]	p.(=)	TG14T5	2	2 M	1.25%	NF	NF	VUS	Not relevant	Not relevant

M: male; F: female; DC: disease-causing; VCC: varying clinical consequences; NF: not found; US: unknown significance; LP: likely pathogenic.

**Table 2:** CFTR genetic status of couples, risk classification and final comments on eligibility for PGT-M. Unless both alleles are reported, all described variants were identified as heterozygous.

Couple Id	Female Partner		Male Partner		Predicted Offspring Phenotype	Indication to Pgt
	Coding Sequence/Protein Change		Coding Sequence/Protein Change			
1	c.3454G>C	p.(Asp1152His)	c.2051_2052delAAinsG	p.(Lys684Thrfs*4)	CF-varying consequences	Eligible
2	c.1521_1523delCTT	p.(Phe508del)	c.579+3A>G	p.(?)	CF	Eligible
3	c.1521_1523delCTT	p.(Phe508del)	c.1040G>C	p.(Arg347Pro)	CF	Eligible
4	c.1521_1523delCTT	p.(Phe508del)	c.1521_1523delCTT	p.(Phe508del)	CF	Eligible
5	c.1521_1523delCTT	p.(Phe508del)	c.3909C>G	p.(Asn1303Lys)	CF	Eligible
6	c.3484C>T	p.(Arg1162*)	c.2051_2052delAAinsG	p.(Lys684Thrfs*4)	CF	Eligible
7	c.1624G>T	p.(Gly542*)	c.1624G>T	p.(Gly542*)	CF	Eligible
8	c.1521_1523delCTT	p.(Phe508del)	c.1585-1G>A	p.(?)	CF	Eligible
9	c.3485G>T	p.(Arg1162Leu)	c.1521_1523delCTT	p.(Phe508del)	CFTR-RD	Eligible
10	c.3230T>C	p.(Leu1077Pro)	c.2051_2052delAAinsG	p.(Lys684Thrfs*4)	CF	Eligible
11	c.1521_1523delCTT	p.(Phe508del)	c.3485G>T	p.(Arg1162Leu)	CFTR-RD	Eligible
12	c.1521_1523delCTT	p.(Phe508del)	c.2249C>T	p.(Pro750Leu)	CF-varying consequences	Eligible
13	c.1521_1523delCTT	p.(Phe508del)	c.1521_1523delCTT	p.(Phe508del)	CF	Eligible
14	c.1521_1523delCTT	p.(Phe508del)	c.1624G>T	p.(Gly542*)	CF	Eligible
15	c.3909C>G	p.(Asn1303Lys)	c.1624G>T	p.(Gly542*)	CF	Eligible
16	c.1521_1523delCTT	p.(Phe508del)	c.3484C>T	p.(Arg1162*)	CF	Eligible
17	c.3484C>T	p.(Arg1162*)	c.1521_1523delCTT	p.(Phe508del)	CF	Eligible
18	c.1521_1523delCTT	p.(Phe508del)	c.3718-2477C>T	p.(?)	CF	Eligible
19	c.3484C>T	p.(Arg1162*)	c.3484C>T	p.(Arg1162*)	CF	Eligible
20	c.3302T>A	p.(Met1101Lys)	c.1766+1G>A	p.(?)	CF	Eligible
21	c.2052dup	p.(Gln685Thrfs*4)	c.1521_1523delCTT	p.(Phe508del)	CF	Eligible
22	c.[1521_1523delCTT];[489+3A>G]	p.[(Phe508del)];[(?)]	c.2657+5G>A	p.(?)	CF	Eligible
23	c.3454G>C	p.(Asp1152His)	c.1521_1523delCTT	p.(Phe508del)	CF-varying consequences	Eligible
24	c.1040G>C	p.(Arg347Pro)	c.350G>A	p.(Arg117His)	CF-varying consequences	Eligible
25	c.2463_2464delTG	p.(Ser821Argfs*4)	c.489+3A>G	p.(?)	CF-varying consequences	Eligible
26	c.3484C>T	p.(Arg1162*)	c.2051_2052delAAinsG	p.(Lys684Thrfs*4)	CF	Eligible
27	c.1521_1523delCTT	p.(Phe508del)	c.579+3A>G	p.(?)	CF	Eligible
28	c.1584+18672A>G	p.(?)	c.489+3A>G	p.(?)	CF	Eligible
29	c.1521_1523delCTT	p.(Phe508del)	c.1521_1523delCTT	p.(Phe508del)	CF	Eligible
30	c.1521_1523delCTT	p.(Phe508del)	c.1521_1523delCTT	p.(Phe508del)	CF	Eligible
31	c.1521_1523delCTT	p.(Phe508del)	c.1521_1523delCTT	p.(Phe508del)	CF	Eligible
32	c.1521_1523delCTT	p.(Phe508del)	c.1040G>C	p.(Arg347Pro)	CF	Eligible
33	c.1585-1G>A	p.(?)	c.2657+5G>A	p.(?)	CF-varying consequences	Eligible
34	c.3889dup	p.(Ser1297Phefs*5)	c.2856G>A	p.(Met952Ile)	CFTR-RD	Eligible
35	c.3454G>C	p.(Asp1152His)	c.3908delA	p.(Asn1303Thrfs*25)	CF-varying consequences	Eligible
36	c.1521_1523delCTT	p.(Phe508del)	c.1521_1523delCTT	p.(Phe508del)	CF	Eligible
37	c.1521_1523delCTT	p.(Phe508del)	c.1521_1523delCTT	p.(Phe508del)	CF	Eligible
38	c.601G>A	p.(Val201Met)	c.1521_1523delCTT	p.(Phe508del)	CFTR-RD	Eligible
39	c.1521_1523delCTT	p.(Phe508del)	c.1521_1523delCTT	p.(Phe508del)	CF	Eligible
40	c.1624G>T	p.(Gly542*)	c.3909C>G	p.(Asn1303Lys)	CF	Eligible
41	c.[4426C>T];[1521_1523delCTT]	p.[(Gln1476*)];[(Phe508del)]	c.2991G>C	p.(Leu997Phe)	CFTR-RD	Eligible
42	c.274-1G>C	p.(?)	c.1624G>T	p.(Gly542*)	CF	Eligible
43	c.3846G>A	p.(Trp1282*)	c.[3154T>G];[1209+28C>T]	p.[(Phe1052Val)];[(?)]	CF-varying consequences	Eligible
44	c.1521_1523delCTT	p.(Phe508del)	c.3484C>T	p.(Arg1162*)	CF	Eligible
45	c.1210-34TG[13];1210-12T[5]	p.(=)	c.1521_1523delCTT	p.(Phe508del)	CF-varying consequences	Eligible
46	c.1521_1523delCTT	p.(Phe508del)	c.3909C>G	p.(Asn1303Lys)	CF	Eligible
47	c.1521_1523delCTT	p.(Phe508del)	c.274-1G>C	p.(?)	CF	Eligible
48	c.3454G>C	p.(Asp1152His)	c.1521_1523delCTT	p.(Phe508del)	CF-varying consequences	Eligible
49	c.1521_1523delCTT	p.(Phe508del)	c.1584+18672A>G	p.(?)	CF	Eligible
50	c.2657+5G>A	p.(?)	c.3194T>C	p.(Leu1065Pro)	CF	Eligible
51	c.1624G>T	p.(Gly542*)	c.328G>A	p.(Asp110Asn)	Uncertain risk	Insufficient evidence/situational
52	c.2787C>T	p.(=)	c.1210-34TG[14];1210-12T[5]	p.(=)	Uncertain risk	Insufficient evidence/situational
53	c.961T>C	p.(Ser321Pro)	c.1521_1523delCTT	p.(Phe508del)	Uncertain risk	Insufficient evidence/situational
54	c.1046C>T	p.(Ala349Val)	c.1521_1523delCTT	p.(Phe508del)	Uncertain risk	Insufficient evidence/situational
55	c.317T>C	p.(Ile106Thr)	c.3484C>T	p.(Arg1162*)	Uncertain risk	Insufficient evidence/situational
56	c.1392+3A>G	p.(?)	c.2900T>C	p.(Leu967Ser)	Uncertain risk	Insufficient evidence/situational
57	c.1521_1523delCTT	p.(Phe508del)	c.1163C>T	p.(Thr388Met)	Uncertain risk	Insufficient evidence/situational
58	c.3909C>G	p.(Asn1303Lys)	c.3854C>A	p.(Ala1285Asp)	Uncertain risk	Insufficient evidence/situational
59	c.1521_1523delCTT	p.(Phe508del)	c.125C>T	p.(Ser42Phe)	Uncertain risk	Insufficient evidence/situational
60	c.316A>T	p.(Ile106Leu)	c.2471G>C	p.(Gly885Ala)	Uncertain risk	Insufficient evidence/situational
61	c.2051_2052delAAinsG	p.(Lys684Thrfs*4)	c.1210-34TG[14];1210-12T[5]	p.(=)	Uncertain risk	Insufficient evidence/situational
62	c.254G>A	p.(Gly85Glu)	c.224G>A	p.(Arg75Gln)	Low risk	No
63	c.1584G>A	p.(=)	c.1521_1523delCTT	p.(Phe508del)	Low risk	No
64	c.1584G>A	p.(=)	c.1585-1G>A	p.(?)	Low risk	No
65	Negative		c.2051_2052delAAinsG	p.(Lys684Thrfs*4)	Low risk	No
66	Negative		c.3718-2477C>T	p.(?)	Low risk	No
67	Negative		c.1521_1523delCTT	p.(Phe508del)	Low risk	No
68	Negative		c.1521_1523delCTT	p.(Phe508del)	Low risk	No
69	c.2051_2052delAAinsG	p.(Lys684Thrfs*4)	Negative		Low risk	No
70	c.3230T>C	p.(Leu1077Pro)	Negative		Low risk	No
71	c.[601G>A;1210-12T[5]]	p.(Val201Met)	Negative		Low risk	No
72	Negative		c.3909C>G	p.(Asn1303Lys)	Low risk	No
73	Negative		c.3718-2477C>T	p.(?)	Low risk	No
74	c.489+3A>G	p.(?)	Negative		Low risk	No
75	c.350G>A	p.(Arg117His)	c.1210-34TG[10];1210-12T[5]	p.(=)	Low risk	No
76	c.[2657+5G>A];[c.3846G>A]	p.[(?)];[(Trp1282*)]	Negative		Low risk	No
77	c.3909C>G	p.(Asn1303Lys)	Negative		Low risk	No
78	Negative		c.1210-34TG[10];1210-12T[5]	p.(=)	Low risk	No
79	Negative		c.1521_1523delCTT	p.(Phe508del)	Low risk	No
80	c.224G>A	p.(Arg75Gln)	c.1584G>A	p.(=)	Low risk	No
81	c.3454G>C	p.(Asp1152His)	c.1210-34TG[10];1210-12T[5]	p.(=)	Low risk	No
82	Negative		c.1521_1523delCTT	p.(Phe508del)	Low risk	No
83	Negative		c.[1210-12T[7];c.[1210-12T[9]]	p.[=];[=]	Low risk	No
84	Negative		c.1210-34TG[10];1210-12T[5]	p.(=)	Low risk	No
85	c.2051_2052delAAinsG	p.(Lys684Thrfs*4)	Negative		Low risk	No
86	c.[1210-12T[7];c.[1210-12T[7]]	p.[=];[=]	Negative		Low risk	No
87	Negative		c.1210-34TG[10];1210-12T[5]	p.(=)	Low risk	No
88	Negative		c.489+3A>G	p.(?)	Low risk	No
89	c.1624G>T	p.(Gly542*)	Negative		Low risk	No
90	Negative		c.1521_1523delCTT	p.(Phe508del)	Low risk	No
91	c.1210-34TG[12];1210-12T[5]	p.(=)	Negative		Low risk	No
92	Negative		c.[1210-12T[7];c.[1210-12T[7]]	p.[=];[=]	Low risk	No

**Table 3:** Predicted response to postnatal treatment with CFTR modulators for the offspring of couples with high or undetermined risk of disease.

Couple Id	Female Partner		Male Partner		Cftr Modulator(S)			Therapy Available in Case of Affected Offspring
	Coding Sequence/Protein Change		Coding Sequence/Protein Change					
1	c.3454G>C	p.(Asp1152His)	c.2051_2052delAAinsG	p.(Lys684Thrfs*4)		T; I	E; T; I	YES
2	c.1521_1523delCTT	p.(Phe508del)	c.579+3A>G	p.(?)	L; I	T; I	E; T; I	YES
3	c.1521_1523delCTT	p.(Phe508del)	c.1040G>C	p.(Arg347Pro)	L; I	T; I	E; T; I	YES
4	c.1521_1523delCTT	p.(Phe508del)	c.1521_1523delCTT	p.(Phe508del)	L; I	T; I	E; T; I	YES
5	c.1521_1523delCTT	p.(Phe508del)	c.3909C>G	p.(Asn1303Lys)	L; I	T; I	E; T; I	YES
6	c.3484C>T	p.(Arg1162*)	c.2051_2052delAAinsG	p.(Lys684Thrfs*4)	No	No	No	NO
7	c.1624G>T	p.(Gly542*)	c.1624G>T	p.(Gly542*)	No	No	No	NO
8	c.1521_1523delCTT	p.(Phe508del)	c.1585-1G>A	p.(?)	L; I	T; I	E; T; I	YES
9	c.3485G>T	p.(Arg1162Leu)	c.1521_1523delCTT	p.(Phe508del)	L; I	T; I	E; T; I	YES
10	c.3230T>C	p.(Leu1077Pro)	c.2051_2052delAAinsG	p.(Lys684Thrfs*4)			E; T; I	NO
11	c.1521_1523delCTT	p.(Phe508del)	c.3485G>T	p.(Arg1162Leu)	L; I	T; I	E; T; I	YES
12	c.1521_1523delCTT	p.(Phe508del)	c.2249C>T	p.(Pro750Leu)	L; I	T; I	E; T; I	YES
13	c.1521_1523delCTT	p.(Phe508del)	c.1521_1523delCTT	p.(Phe508del)	L; I	T; I	E; T; I	YES
14	c.1521_1523delCTT	p.(Phe508del)	c.1624G>T	p.(Gly542*)	No	No	No	NO
15	c.3909C>G	p.(Asn1303Lys)	c.1624G>T	p.(Gly542*)	No	No	No	NO
16	c.1521_1523delCTT	p.(Phe508del)	c.3484C>T	p.(Arg1162*)	L; I	T; I	E; T; I	YES
17	c.3484C>T	p.(Arg1162*)	c.1521_1523delCTT	p.(Phe508del)	L; I	T; I	E; T; I	YES
18	c.1521_1523delCTT	p.(Phe508del)	c.3718-2477C>T	p.(?)	L; I	T; I	E; T; I	YES
19	c.3484C>T	p.(Arg1162*)	c.3484C>T	p.(Arg1162*)	No	No	No	NO
20	c.3302T>A	p.(Met1101Lys)	c.1766+1G>A	p.(?)	No	No	E; T; I	YES
21	c.2052dup	p.(Gln685Thrfs*4)	c.1521_1523delCTT	p.(Phe508del)	L; I	T; I	E; T; I	YES
22	c.[1521_1523delCTT];[489+3A>G]	p.[(Phe508del)];[(?)]	c.2657+5G>A	p.(?)	L; I	T; I	E; T; I	YES
23	c.3454G>C	p.(Asp1152His)	c.1521_1523delCTT	p.(Phe508del)	L; I	T; I	E; T; I	YES
24	c.1040G>C	p.(Arg347Pro)	c.350G>A	p.(Arg117His)	I	I; T	E; T; I	YES
25	c.2463_2464delTG	p.(Ser821Argfs*4)	c.489+3A>G	p.(?)	No	No	No	NO
26	c.3484C>T	p.(Arg1162*)	c.2051_2052delAAinsG	p.(Lys684Thrfs*4)	No	No	No	NO
27	c.1521_1523delCTT	p.(Phe508del)	c.579+3A>G	p.(?)	L; I	T; I	E; T; I	YES
28	c.1584+18672A>G	p.(?)	c.489+3A>G	p.(?)	No	No	No	NO
29	c.1521_1523delCTT	p.(Phe508del)	c.1521_1523delCTT	p.(Phe508del)	L; I	T; I	E; T; I	YES
30	c.1521_1523delCTT	p.(Phe508del)	c.1521_1523delCTT	p.(Phe508del)	L; I	T; I	E; T; I	YES
31	c.1521_1523delCTT	p.(Phe508del)	c.1521_1523delCTT	p.(Phe508del)	L; I	T; I	E; T; I	YES
32	c.1521_1523delCTT	p.(Phe508del)	c.1040G>C	p.(Arg347Pro)	L; I	T; I	E; T; I	YES
33	c.1585-1G>A	p.(?)	c.2657+5G>A	p.(?)	No	No	No	NO
34	c.3889dup	p.(Ser1297Phefs*5)	c.2856G>A	p.(Met952Ile)	No	No	No	NO
35	c.3454G>C	p.(Asp1152His)	c.3908delA	p.(Asn1303Thrfs*25)	No	T; I	E; T; I	YES
36	c.1521_1523delCTT	p.(Phe508del)	c.1521_1523delCTT	p.(Phe508del)	L; I	T; I	E; T; I	YES
37	c.1521_1523delCTT	p.(Phe508del)	c.1521_1523delCTT	p.(Phe508del)	L; I	T; I	E; T; I	YES
38	c.601G>A	p.(Val201Met)	c.1521_1523delCTT	p.(Phe508del)	L; I	T; I	E; T; I	YES
39	c.1521_1523delCTT	p.(Phe508del)	c.1521_1523delCTT	p.(Phe508del)	L; I	T; I	E; T; I	YES
40	c.1624G>T	p.(Gly542*)	c.3909C>G	p.(Asn1303Lys)	No	No	No	NO
41	c.[4426C>T];[1521_1523delCTT]	p.[(Gln1476*)];[(Phe508del)]	c.2991G>C	p.(Leu997Phe)	L; I	T; I	E; T; I	YES
42	c.274-1G>C	p.(?)	c.1624G>T	p.(Gly542*)	No	No	No	NO
43	c.3846G>A	p.(Trp1282*)	c.[3154T>G];[1209+28C>T]	p.[(Phe1052Val)];[(?)]	I	T; I	E; T; I	YES
44	c.1521_1523delCTT	p.(Phe508del)	c.3484C>T	p.(Arg1162*)	L; I	T; I	E; T; I	YES
45	c.1210-34TG[13];1210-12T[5]	p.(=)	c.1521_1523delCTT	p.(Phe508del)	L; I	T; I	E; T; I	YES
46	c.1521_1523delCTT	p.(Phe508del)	c.3909C>G	p.(Asn1303Lys)	L; I	T; I	E; T; I	YES
47	c.1521_1523delCTT	p.(Phe508del)	c.274-1G>C	p.(?)	L; I	T; I	E; T; I	YES
48	c.3454G>C	p.(Asp1152His)	c.1521_1523delCTT	p.(Phe508del)	L; I	T; I	E; T; I	YES
49	c.1521_1523delCTT	p.(Phe508del)	c.1584+18672A>G	p.(?)	L; I	T; I	E; T; I	YES
50	c.2657+5G>A	p.(?)	c.3194T>C	p.(Leu1065Pro)	No	No	No	NO
51	c.1624G>T	p.(Gly542*)	c.328G>A	p.(Asp110Asn)	No	No	No	NO
52	c.2787C>T	p.(=)	c.1210-34TG[14];1210-12T[5]	p.(=)	No	No	No	NO
53	c.961T>C	p.(Ser321Pro)	c.1521_1523delCTT	p.(Phe508del)	L; I	T; I	E; T; I	YES
54	c.1046C>T	p.(Ala349Val)	c.1521_1523delCTT	p.(Phe508del)	L; I	T; I	E; T; I	YES
55	c.317T>C	p.(Ile106Thr)	c.3484C>T	p.(Arg1162*)	No	No	No	NO
56	c.1392+3A>G	p.(?)	c.2900T>C	p.(Leu967Ser)	No	No	No	NO
57	c.1521_1523delCTT	p.(Phe508del)	c.1163C>T	p.(Thr388Met)	L; I	T; I	E; T; I	YES
58	c.3909C>G	p.(Asn1303Lys)	c.3854C>A	p.(Ala1285Asp)	No	No	No	NO
59	c.1521_1523delCTT	p.(Phe508del)	c.125C>T	p.(Ser42Phe)	L; I	T; I	E; T; I	YES
60	c.316A>T	p.(Ile106Leu)	c.2471G>C	p.(Gly885Ala)	No	No	No	NO
61	c.2051_2052delAAinsG	p.(Lys684Thrfs*4)	c.1210-34TG[14];1210-12T[5]	p.(=)	No	No	No	NO

L: lumacaftor; E: elexacaftor; T: tezacaftor; I: ivacaftor.

## Data Availability

The data presented in this study are available on request to the corresponding author.
